# Prognostic values of baseline cortisol levels and neutrophil to lymphocyte ratio in COVID-19

**DOI:** 10.5937/jomb0-38533

**Published:** 2023-08-25

**Authors:** Sengel Buket Erturk, Tigen Elif Tukenmez, Can Ilgin, Volkan Korten, Zekaver Odabasi

**Affiliations:** 1 Marmara University, Pendik Training and Research Hospital, Department of Infectious Disease and Clinical Microbiology, Istanbul, Turkey; 2 Marmara University, Pendik Training and Research Hospital, Department of Public Health, Istanbul, Turkey

**Keywords:** cortisol, COVID-19, neutrophil to lymphocyte ratio, prediction, severity, kortizol, COVID-19, odnos neutrofila i limfocita, predviđanje, težina

## Abstract

**Background:**

The prediction of disease severity in COVID19 could be a valuable tool for providing early treatment and reducing mortality. We aimed to evaluate the predictor value of baseline cortisol values on disease severity and assess the correlation between the neutrophil to lymphocyte ratio (NLR) and cortisol levels.

**Methods:**

In this retrospective study, we compared the prognostic value of baseline NLR, morning cortisol, ferritin, and C-reactive protein (CRP) levels among patients with severe and non-severe COVID-19. The association was assessed with Spearman's correlation.

**Results:**

37.7% of the patients (n=63) had severe disease, and their baseline cortisol levels were higher than those in the non-severe group (522 nmol/L vs 380.7 nmol/L, p=0.011). The baseline cortisol level and NLR had area under the curve (AUC) values of 0.62 (95% confidence interval CI 0.53-0.71) and 0.70 (CI 95% 0.62-0.78) for the prediction of severe COVID-19, respectively. Severe disease was predicted in patients with a baseline cortisol cutoff ≥ 522 nmol/L with a specificity of 75.0%, a sensitivity of 50.79%. The cutoff value for the NLR on day 1 was ≥ 6.2, with a specificity of 93.27% and a sensitivity of 32.79%. Baseline cortisol levels showed a significant weakmoderate positive correlation with the NLR and levels of CRP and ferritin on day 1 (r=0.33, r=0.29, r=0.28, respectively, p<0.001 for all).

**Conclusions:**

The baseline cortisol level in COVID-19 patients is a good predictive marker for disease severity and non-inferior to the NLR. However, it is inferior to CRP and ferritin.

## Abstract (sr)

**Uvod:**

Predviđanje težine bolesti kod COVID-19 moglo bi biti dragoceno sredstvo za pružanje ranog lečenja i smanjenje smrtnosti. Cilj nam je bio da procenimo prediktorsku vrednost osnovnih vrednosti kortizola na ozbiljnosti bolesti i procenimo korelaciju između odnosa neutrofila i limfocita (NLR) i nivoa kortizola.

**Metode:**

U ovoj retrospektivnoj studiji uporedili smo prognostičku vrednost osnovnog nivoa NLR, jutarnjeg kortizola, feritina i C-reaktivnog proteina (CRP) među pacijentima sa teškim i ne-teškim COVID-19. Povezanost je procenjena Spearmanovom korelacijom.

**Rezultati:**

37,7% pacijenata (n=63) je imalo tešku bolest, a njihovi osnovni nivoi kortizola su bili viši od onih u grupi koja nije bila teška (522 nmol/L naspram 380.7 nmol/L, p=0,011). Osnovni nivo kortizola i NLR su imali vrednosti površine ispod krive (AUC) od 0,62 (95% interval poverenja CI 0,53-0,71) i 0,70 (CI 95% 0,62-0,78) za predviđanje teškog COVID-19, respektivno. Predviđena je teška bolest kod pacijenata sa početnom graničnom vrednostom kortizola ≥ 522 nmol/L sa specifičnošću od 75,0%, osetljivošću od 50,79%. Granična vrednost za NLR prvog dana bila je ≥ 6,2, sa specifičnošću od 93,27% i osetljivošću od 32,79%. Osnovni nivoi kortizola su pokazali značajnu slabu-umerenu pozitivnu korelaciju sa NLR i nivoima CRP i feritina prvog dana (r=0,33, r=0,29, r=0,28, respektivno, p<0,001 za sve).

**Zaključak:**

Osnovni nivo kortizola kod pacijenata sa COVID-19 je dobar prediktivni marker za ozbiljnost bolesti i nije inferioran u odnosu na NLR. Međutim, on je inferioran u odnosu na CRP i feritin.

## Introduction

The majority of coronavirus disease 2019 (COVID-19) patients have asymptomatic or mild disease, while approximately 10-15% have a severe disease [1] [2]. Identifying poor prognosis predictors is important when choosing different treatment modalities. Many prognostic factors, such as the levels of interleukin 6 (IL-6), C-reactive protein (CRP), ferritin, procalcitonin, and D-dimer and the lymphocyte percentage or count, have been previously described in the literature [3] [4] [5] [6]. Recently, analyses of the neutrophil-to-lymphocyte ratio (NLR) have been shown to be a strong prognostic factor [7] [8] [9] [10] [11].

Sparse information exists on the prognostic value of basal cortisol levels in COVID-19 patients. Previous studies demonstrated that severe acute respiratory syndrome related to COVID-19 (SARS-CoV-2) might trigger a stress response and activate the hypothalamic-pituitary-adrenal (HPA) axis [12] [13]. Increased secretion of cortisol from the adrenals causes the migration of lymphocytes from the peripheral circulation and the inhibition of neutrophil apoptosis, thereby increasing the NLR [14]. A recently published study suggested that high baseline cortisol levels are associated with higher mortality and decreased survival in COVID-19 patients [15] [16] [17].

In this retrospective study, we aimed to evaluate the relationship between baseline cortisol levels and prognosis in COVID-19 patients, in addition to the correlations of baseline cortisol levels with the NLR and other prognostic factors.

## Materials and methods

This retrospective study was performed between March 27 and May 13, 2020 (the last follow-up was on June 3) at Marmara University Pendik Research and Training Hospital. ≥ The inclusion criteria were: 1. Patients aged ≥ 18 years; 2. Positive real-time polymerase chain reaction (RT-PCR) results for SARS-CoV-2; 3. Patients hospitalized with symptoms due to COVID-19, regardless of the presence of hypoxia. Hypoxia was defined as saturation of oxygen <94% on room air. The exclusion criteria were: 1. Patients requiring intubation on admission; 2. Patients have been using topical and systemic corticosteroids for any other reason. The patients were followed for up to 14 days after discharge. A waiver of informed consent was issued by the Marmara University School of Medicine Institutional Ethical Review Board (Reference number: 09.2021.300).

Sex, age, number of comorbidities (hypertension, diabetes mellitus, chronic obstructive pulmonary disease (COPD), asthma, immunosuppression, cardiovascular system disease, chronic renal and liver disease), serum inflammatory markers (neutrophil and lymphocyte counts and percentages, NLR, CRP, ferritin), baseline cortisol level (within 48 hours of admission), and clinical outcomes (discharge or inhospital mortality) were obtained from computerbased patient records. The baseline cortisol levels were assessed in the morning. The laboratory parameters were checked on admission and every other day thereafter except for the cortisol levels which were only checked once on admission.

During the study period, none of the patients were started on steroids as the evidence that showed a benefit of such treatment did not exist at the time of this study interval [18].

According to the National Institutes of Health (NIH) classification, severe cases were defined by saturation of oxygen <94% on room air at sea level, a ratio of arterial PaO_2_/FiO_2_ <300 mm Hg, respiratory rate >30 breaths/min, or lung infiltrates >50% [19]. Patients who met these criteria on admission or during their hospital stay were diagnosed with severe COVID-19.

### Statistical analysis

In this descriptive study, we compared the demographic, clinical characteristics, and laboratory values of the severe and non-severe COVID-19 patients. The assumption of normality for numerical variables was tested with histograms, normal quantile plots, and Kolmogorov-Smirnov tests. Numerical variables without a normal distribution are reported as the median, interquartile range (IQR), and minimum and maximum values. Categorical variables are reported as frequencies and percentages. The differences in the distributions of numerical variables between severe and non-severe COVID-19 patients were analyzed with Mann-Whitney U tests. The comparisons of categorical variables were performed with either the chi-square test or Fisher's exact test. The correlations between numerical variables were calculated with Spearman’s correlation test, and rho and p values are reported. The area under the curve (AUC) values for different laboratory tests for the prediction of severe COVID-19 were calculated with nonparametric ROC analysis and are presented with 95% confidence intervals [Cls] and standard errors. The AUC values of ROC curves were compared with the roccomp module, and AUC values with their standard errors and 95% CIs and p values are reported. The sensitivity, specificity,correct classification percentage, and positive and negative likelihood ratios (LRs) are reported for selected laboratory methods. The databases were created with Microsoft Excel 2007, and all statistical analyses were performed with Stata 15.1. A p-value less than 0.05 was considered statistically significant.

## Results

We included 167 hospitalized adult patients with positive RT-PCR results for SARS-CoV-2 in our study. The median age of the patients was 54 years (interquartile range [IQR], 20), and 87 (52.1%) were female. Of the 167 patients, 63 (37.7%) patients had severe disease, and 104 (62.2%) had non-severe disease. One hundred ten patients had at least one comorbidity. The most common comorbidities were hypertension (n=64, 38.3%), diabetes (n=47,28.1%), and cardiovascular disease (n=24, 14.3%). Only 6 patients died during the study period

The overall median baseline cortisol level was 412.4 nmol/L (IQR, 282.8). The NLR was 3 (IQR, 3.13), 2.6 (IQR, 2.61), and 2.92 (IQR, 2.36) on days 1, 3, and 5, respectively. Baseline cortisol levels were significantly higher in the severe group than in the non-severe group (522 nmol/L vs 380.7 nmol/L, p=0.011). The NLR and ferritin values were also significantly higher severe group for three measurements taken every other day (p <0.001 for all). CRP values were higher in the severe group for the first two measurements (p <0.001). The characteristics of the patients are summarized in Table 1.

**Table 1:** Characteristics of the patients. Abbreviations: CRP, C-reactive protein; IQR, interquartile range; NLR, neutrophil to lymphocyte ratio.

Patient Characteristic	Statistics	Total (n=167)	Severe (n=63)	Non-Severe (n=104)	p
Age, years	Median (IQR)	54 (20)	58 (19)	54 (23.5)	0.008
	Min-Max	22–96	40–96	22–93	
Sex (Male)	n (%)	80 (47.90)	39 (61.90)	41 (39.42)	0.005
Comorbidities					
Comorbidity	n (%)	110 (65.87)	41 (65.08)	69 (66.35)	0.867
Hypertension	n (%)	64 (38.32)	25 (39.68)	39 (37.5)	0.779
Diabetes mellitus	n (%)	47 (28.14)	19 (30.16)	28 (26.92)	0.652
Cardiovascular disease	n (%)	24 (14.37)	12 (19.05)	12 (11.54)	0.180
Chronic liver disease	n (%)	5 (2.99)	3 (4.76)	2 (1.92)	0.367
Immunosuppression	n (%)	7 (4.19)	5 (7.94)	2 (1.92)	0.105
Chronic lung disease	n (%)	8 (4.79)	6 (9.52)	2 (1.92)	0.054
Asthma	n (%)	17 (10.18)	2 (3.17)	15 (14.42)	0.019
Chronic renal disease	n (%)	9 (5.39)	3 (4.76)	6 (5.77)	1.000
Laboratory					
Baseline cortisol^a^nmol/L	Median (IQR)	412.4 (282.8)	522 (325.8)	380.7 (241.4)	0.011
	Min-Max	50.4–1683	50.4–1683	75.8–993.2	
NLR (day 1)	Median (IQR)	3.00 (3.13)	4.4 (4.42)	2.54 (2.64)	<0.001
	Min-Max	0.13–32.50	1.07–32.5	0.13–27	
NLR (day 3)	Median (IQR)	2.60 (2.62)	4.00 (4.75)	2.24 (1.76)	<0.001
	Min-Max	0.47–54.55	0.47–54.55	0.83–9.40	
NLR (day 5)	Median (IQR)	2.92 (2.36)	3.45 (3.06)	2.52 (1.60)	<0.001
	Min-Max	0.26–34.60	1.41–34.60	0.26-14.54	
Ferritin (day 1), μg/L	Median (IQR)	137.0 (202.0)	220 (353)	99 (135.5)	<0.001
	Min-Max	1.80–6378.0	5.2–6378	1.8–2403	
Ferritin (day 3), μg/L	Median (IQR)	186.0 (350.0)	325.5 (613.5)	116.5 (166)	<0.001
	Min-Max	2.0–7051.0	6.7–7051	2–1708	
Ferritin (day 5), μg/L	Median (IQR)	219.0 (250.0)	282.5 (358)	167 (216.5)	<0.001
	Min-Max	3.0–4192.0	7.3–4192	3.0–2887	
CRP (day 1), mg/L	Median (IQR)	23.0 (48.0)	46 (90)	15 (28.86)	<0.001
	Min-Max	0.90–374.0	3–374	0.9–177	
CRP (day 3), mg/L	Median (IQR)	34.0 (84.0)	75.5 (136)	20 (43.75)	<0.001
	Min-Max	0.5–279.0	0.8–279	0.5–221	
CRP (day 5), mg/L	Median (IQR)	23.0 (48.0)	29 (61)	16.9 (31)	0.107
	Min-Max	0.60–304.0	0.6–303	0.9–304	

**Figure 1:**
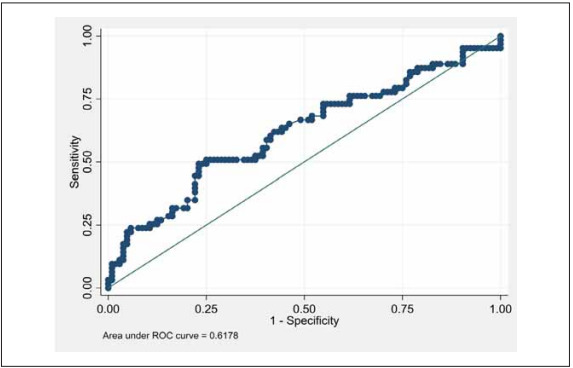
AUC graph for diagnostic performance of baseline cortisol measurement on severe COVID-19.

The baseline cortisol level had an AUC value of 0.62 (95% CI 0.53 to 0.71) for the prediction of severe COVID-19 (Table 2, Figure 1, Supplementary Table 1). The threshold for the baseline cortisol value for the prediction of severe disease was selected as 522 nmol/L and greater, with a specificity of 75.0%, a sensitivity of 50.79%, and a correct classification percentage of 65.87%. This threshold has a positive LR of 2.03 and a negative LR of 0.66.

**Table 2:** Correlations between baseline cortisol and laboratory values on day 1. Abbreviations: CRP, C-reactive protein; NLR, neutrophil to lymphocyte ratio

Laboratory value	rho	p
NLR	0.3269	<0.001
CRP	0.2946	<0.001
Ferritin	0.2816	<0.001

A quarter of patients with non-severe cases (n=26; 25.0%) had baseline cortisol values above the threshold; compared to 32 (50.79%) patients in the severe group. In our patient population, this cortisol threshold had a positive predictive value of 55.2% (95% CI: 41.5%-68.3%) and a negative predictive value of 71.6% (95% CI: 62.1%-79.8%) for a prevalence of 38% (95% CI: 30%-45.5%) (Supplementary Table 1).

The duration between the hospitalization and the development of symptoms had a median value of 5 (IQR=4.0) and ranged between 0 and 60 days. The correlation between the baseline cortisol level and this duration was statistically non-significant and negligible (r=0.08; p=0.31).

**Figure 2:**
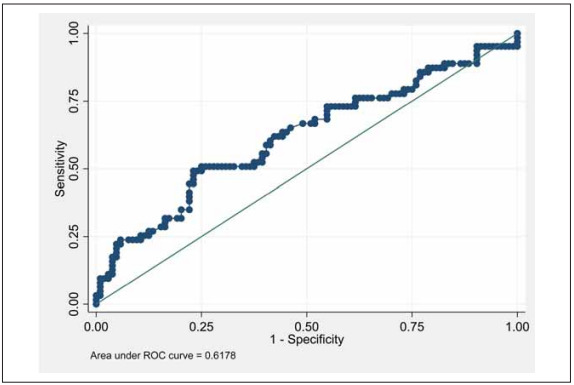
AUC graph for diagnostic performance of day 1 NLR value on severe COVID-19.

The NLR on day 1 had an AUC value of 0.70 (95% CI 0.62 to 0.78) for the prediction of severe COVID-19 (Table 2, Figure 2, Supplementary Table 2). The comparisons of AUC curves for various laboratory parameters for the prediction of severe COVID-19 are shown in Table 2. We selected a cutoff value for the NLR of ≥ 6.2 on day 1, which had a specificity and sensitivity of 93.27% and 32.79%, respectively. The correct classification percentage was 70.91%. The positive and negative LRs were 4.87 and 0.72, respectively. In our patient population, this NLR threshold had a positive predictive value of 74.1% (95% CI 53.7% to 88.9%) and a negative predictive value of 70.3% (95% CI 61.9% to 77.8%) for a prevalence of 37% (95% CI 30% to 44.8%) (Supplementary Table 2).

Baseline cortisol levels had a significant weakmoderate positive correlation with the NLR, CRP, and ferritin levels on day 1 (r=0.33, r= 0.29, r= 0.28, respectively, p<0.001 for all, illustrated in Table 2).

There was no statistically significant difference between the AUC values for baseline cortisol (AUC=0.62; 95% CI 0.53-0.71) and the day 1 NLR (AUC=0.70; 95% CI 0.62-0.78) for the prediction of severe COVID-19 (p=0.14). However, baseline cortisol was inferior to CRP and ferritin as a predictive factor (p=0.03 for all as illustrated in Table 3).

**Table 3:** Comparisons of different laboratory measurements for the prediction of severe COVID-19. Abbreviations: CRP, C-reactive protein; NLR, neutrophil to lymphocyte ratio

	Observation	AUC	Std. Err.	[95% Conf. Interval]		p
Basal cortisol	165	0.62	0.05	0.53	0.71	0.14
NLR (day 1)	165	0.70	0.04	0.62	0.78	
Basal cortisol	165	0.62	0.05	0.53	0.71	0.03
CRP (day 1)	165	0.75	0.04	0.66	0.83	
Basal cortisol	158	0.61	0.05	0.52	0.71	0.03
Ferritin (day 1)	158	0.73	0.04	0.65	0.81	

## Discussion

Although most COVID-19 patients have a mild clinical course, some can deteriorate within 7-14 days [20]. Therefore, predicting severe disease is critical to provide appropriate supportive care, enable early access to intensive care units (ICUs), and reduce mortality. Recent studies show, in addition to wellknown factors such as IL-6, CRP, and ferritin, the NLR is also a valuable predictive factor for severe COVID-19 [7] [8] [9] [10] [11] [21]. Liu et al. [7] showed high NLR (3.13), is strongly associated with developing critical disease. In another multi-center retrospective study, the NLR was found to be an independent prognostic tool for progression to critical disease [8]. In our study, the median NLR values of severe group on admission and on days 3 and 5 were significantly higher than those in patients with non-severe cases. An NLR value of ≥ 6.2 on admission was found to be predictive of progression to severe COVID-19.

In addition to the NLR, in a recent study, Tan et al. showed that baseline cortisol levels were higher in patients with COVID-19 than in those without COVID-19 [15]. They determined the optimal cutoff for cortisol in patients with COVID-19 to be 744 nmol/L and showed shorter median survival in patients whose values were greater than this level. Although they found that increased baseline cortisol levels, CRP levels, and NLRs were predictive of acute mortality, they did not report the patients' disease severity. We determined the optimal cutoff for the cortisol value for the prediction of severe cases of COVID-19 to be 522 nmol/L and greater, which is different from the value reported in the study by Tan et al. [15]. When we assessed the correlations of baseline cortisol levels with other laboratory parameters, we found significant weak-moderate positive correlations with the NLR, CRP, and ferritin. Although not statistically significant, the NLR had a better AUC value for the prediction of severe COVID-19 than the baseline cortisol level. The pooled AUC value of the NLR for the prediction of severe disease was reported to be as high as 0.85 (95% CI 0.81-0.88), according to a meta-analysis by Li et al. [9], that includes 13 studies and 1579 patients. We showed that baseline cortisol values are as good as the NLR but inferior to the CRP and ferritin for the prediction of severe disease. Therefore, baseline cortisol levels may be used as an independent prognostic marker for the prediction of disease severity. Further studies are needed to compare the prognostic performances of basal cortisol levels and NLRs for the prediction of severe COVID-19.

One of the limitations of our study is that cortisol levels were measured once within 48 hours of admission. Second, we were unable to evaluate the plasma adrenocorticotropic hormone (ACTH) concentration and clarify whether patients with low cortisol levels had adrenal insufficiency. Third, the effect of baseline cortisol levels on mortality couldn't be evaluated due to the small number of patients who died. Fourth, this study was not designed as a case-control study, and confounding factors may have affected the results.

## Conclusion

In conclusion, a high baseline cortisol level may be used as a predictive marker for severe disease in COVID-19 patients. However, further studies are warranted to prove this finding.

## Dodatak

### Acknowledgments

### Funding sources

No funding sources.

### Ethical approval

The study protocol was approved by the local ethics committee of Marmara University School of Medicine (Reference number: 09.2021.300).

### Author Contributions

Buket Erturk Sengel; collected the data, wrote the manuscript, and designed the tables. Elif Tukenmez Tigen; collected and contributed the data, Can Ilgin; analysed and interpreted the data and designed the tables, Volkan Korten; revising it critically for intellectual content, Zekaver Odabasi; conception and design of the study. All authors have read and approved the final manuscript.

### Conflict of interest statement

All the authors declare that they have no conflict of interest in this work.
